# *CytoJournal*'s move to fund Open Access

**DOI:** 10.1186/1742-6413-2-3

**Published:** 2005-02-10

**Authors:** Vinod B Shidham, Anthony F Cafaro, Barbara F Atkinson

**Affiliations:** 1 Co-Editor-in-chief, CytoJournal, Medical College of Wisconsin, Milwaukee, WI, USA; 2 Assiatant editor, CytoJournal, Medical College of Wisconsin, Milwaukee, WI, USA; 3 Co-Editor-in-chief, CytoJournal, Kansas University Medical Center, Kansas City, KS, USA

**Keywords:** Cytopathology, cytology, internet, Open Access, peer reviewed research, online

## Abstract

*CytoJournal *is published by an independent publisher BioMed Central, which is committed to ensuring that the peer-reviewed biomedical research is Open Access. Since its launch, BioMed Central has graciously supported the processing of all the articles published during *CytoJournal'*s first 6 months. However, for long term viability, *CytoJournal *has to achieve financial viability to support publication expenses. From 1st March, 2005, authors will be asked by the publisher to pay a flat article-processing charge. This editorial discusses how a significant proportion of authors may not have to pay this fee directly under a variety of different mechanisms such as institutional and society memberships with BioMed Central.

## Introduction

*CytoJournal *is published by BioMed Central, an independent publisher committed to ensuring peer-reviewed biomedical research is Open Access – it is universally and freely available online to everyone, its authors retain copyright, and it is archived in at least one internationally recognised free repository [1]. *CytoJournal *however, has taken this further, by making all its content Open Access.

Since its launch in August 2004, BioMed Central has graciously supported the processing of all the articles published during *CytoJournal*'s first 6 months. However, as we move forward it is crucial that *CytoJournal *develops its own financial viability, at least to support publication expenses. Thus, from 1^st ^March, 2005, authors will be asked by the publisher to pay a flat £330 (approximately US $600/€500) article-processing charge (APC) [2]. As explained below, a significant proportion of authors may not have to pay the fee directly. This is because the APC can be covered/waived under a variety of different mechanisms such as institutional and society memberships with BioMed Central [2].

Please note that authors submitting any article online to CytoJournal before 1^st ^March, 2005 will not have to pay an APC, even if it is accepted for publication after peer review.

## Problems with the traditional publishing model

Traditionally, readers pay to access articles, either through subscriptions or by paying a fee each time they download an article. Escalating journal subscriptions have resulted in libraries subscribing to fewer journals [3], and the range of articles available to readers is therefore limited. Although traditional journals publish authors' work for free (unless there are page or colour charges), having to pay to access articles limits how many people can read, use and cite them. This compromises the scientific impact of the publication and overall visibility of the researchers work.

## Definition of Open Access

*CytoJournal*'s Open Access policy changes the way in which articles are published. First, all articles become freely and universally accessible online, and so an author's work can be read by anyone at no cost. Second, the authors hold copyright for their work and grant anyone the right to reproduce and disseminate the article, provided that it is correctly cited and no errors are introduced [1]. Third, a copy of the full text of each article is permanently archived in an online repository separate from the journal. *CytoJournal*'s articles are archived in PubMed Central [4], the US National Library of Medicine's full-text repository of life science literature, and also in repositories at the University of Potsdam [5] in Germany, at INIST [6] in France, and in e-Depot [7]- the National Library of the Netherlands' digital archive of all electronic publications.

## Benefits of Open Access

Open Access has four broad benefits for science and the general public. First, authors are assured that their work is disseminated to the widest possible audience, given that there are no barriers to access it. This is accentuated by the authors being free to reproduce and distribute their work, for example by placing it on their institution's website. They can still use the published material for any future plans including book chapters, monograms, and presentation after due acknowledgement about its original publication. It has been shown that free online articles are more highly cited because of their easier availability [8]. Second, the information available to researchers will not be limited by what their library can afford, and the widespread availability of articles will enhance literature searching [9]. Third, the results of publicly funded research will be accessible to all taxpayers and not just those with access to a library with a subscription. Note that this public accessibility is becoming a legal requirement for many countries and organizations [10]. Fourth, a country's economy will not influence its scientists' ability to access articles because resource-poor countries (and institutions) will be able to read the same material as wealthier ones (although creating access to the internet is another matter [11]).

Open Access to original peer-reviewed scientific information to both the general public and experts alike has many benefits. It can affect and shape the public opinion. In the long run the availability of such information in the field of cytopathology will translate into further progress in cytopathology and maximize its benefits to various fields of life sciences.

## Description of APC payment

APCs will allow continued Open Access to all of *CytoJournal*'s articles. Authors are asked to pay a flat payment of £330, during 2005, if their article is accepted for publication [2]. Waiver requests will be considered on a case-by-case basis, by the Editor-in-Chief. Authors can circumvent the charge by getting their institution to become a 'member' of BioMed Central, whereby the annual membership fee covers the APCs for all authors at that institution for that year [12]. Current members include numerous institutions in USA, NHS England, the World Health Organization, the US National Institutes of Health, and all UK universities [12]. Biomed Central has also opened a new avenue which allows membership to various societies such as different cytology, cytopathology, and pathology societies all over the world. The members of these societies could publish in *CytoJournal *without incurring an APC. We strongly recommend readers to approach their respective societies to become members under this provision.

No charge is made for articles that are rejected after peer review. Many funding agencies have also realized the importance of Open Access publishing and have specified that their grants may be used directly to pay APCs [13].

As another avenue, Cytopathology-Foundation, Inc , a non-profit organization, is looking for help from appropriate organisations to support the publication cost in *CytoJournal*, so that ultimately our journal will be an Open Access journal free of any financial burden for all *CytoJournal *authors. We appreciate any help in this matter to achieve this goal. You may contact by e-mail: cytojournal@mcw.edu to extend the support.

## What the APC covers

The APC pays for the manuscript publication process. It allows the article to be freely and universally accessible in various formats online, and for the processes required for inclusion in PubMed and archiving in PubMed Central, e-Depot, Potsdam and INIST. Although some authors may consider £330 expensive, it must be remembered that *CytoJournal *does not levy additional page or color charges on top of this fee, which can easily exceed £330 with conventional journals. As the entire manuscript with color PDF file is freely available on-line, authors do not have to stock reprints and spend mailing charges to share the publication with their peers. The PDF files can be directly downloaded by the interested colleague/researcher or the author to share the PDF file through e-mail at the click of the mouse!

With reference to conventional journals it is usually stated in instructions to authors that- 'this journal does not reproduce color illustrations unless the cost of such reproduction is subsidized by the author and agreed upon in advance'. Restricting the number of images published in a manuscript or publishing them as black & white would significantly compromise the publication standard we hope to maintain for a morphology based discipline such as cytopathology. With *CytoJournal*, the article being online only, any number of color figures and photographs can be included at no extra cost. In addition, *CytoJournal *being a web based publication, a small video clip could also be included as part of publication without extra cost. This provision to include video clip(s) is an excellent opportunity to improve the clarity of the details to the readers about dynamic processes such as procedures and methodologies [14].

## Free versus Open Access

Although several journals now offer free access to their articles online, this is different from Open Access (as defined by the Bethesda Statement [15]). These journals often delay free access for 6-12 months, and even when the full text is available, readers are not allowed to reproduce and/or disseminate the work because of restrictions imposed by the copyright policy. That said, *CytoJournal *is not alone in the move to Open Access funded by APCs. The British Medical Journal has recently announced that it cannot continue to provide free access to its website [16] and is considering various sources of revenue, including APCs [17]. Also, the Public Library of Science has set up new Open Access journals, and have elected to set APCs of US$1500 for each accepted article [18]. Given that the Public Library of Science has used television advertising to promote journals [10], the high profile of these journals will raise awareness of Open Access and encourage researchers in all disciplines to understand and accept Open Access, with APCs as an acceptable method to fund it.

## Conclusion

By providing a forum for Open Access, APCs will enable *CytoJournal *to serve the pathology, cytopathology, and entire medical community. We believe this change will benefit and aid scientific research in general. We hope you will support this progress by submitting your next article to *CytoJournal*.

## Abbreviations

APC = article-processing charge.

## Competing interests

At *CytoJournal*, the work of the Editors-in-Chief, the Editorial Board, and of all invited outside peer reviewers is entirely voluntary, without tangible remuneration of any kind. Our goal is publication and dissemination of the highest quality literature and research in cytopathology and related areas. Our (intangible) rewards are in the achievement of these goals. Any decisions about manuscripts are based entirely on the quality of the scientific content, and not on the ability of authors to pay article-processing charges.
